# Therapeutic Monoclonal Antibodies and Antibody Products: Current Practices and Development in Multiple Myeloma

**DOI:** 10.3390/cancers12010015

**Published:** 2019-12-19

**Authors:** Francesca Bonello, Roberto Mina, Mario Boccadoro, Francesca Gay

**Affiliations:** Myeloma Unit, Division of Hematology, University of Torino, Azienda Ospedaliero-Universitaria Città della Salute e della Scienza di Torino, 10126 Torino, Italy

**Keywords:** multiple myeloma (MM), immunotherapy, monoclonal antibodies (mAbs), antibody products, B cell maturation antigens (BCMAs), bispecific T cell engagers (BiTEs^®^)

## Abstract

Immunotherapy is the latest innovation for the treatment of multiple myeloma (MM). Monoclonal antibodies (mAbs) entered the clinical practice and are under evaluation in clinical trials. MAbs can target highly selective and specific antigens on the cell surface of MM cells causing cell death (CD38 and CS1), convey specific cytotoxic drugs (antibody-drug conjugates), remove the breaks of the immune system (programmed death 1 (PD-1) and PD-ligand 1/2 (L1/L2) axis), or boost it against myeloma cells (bi-specific mAbs and T cell engagers). Two mAbs have been approved for the treatment of MM: the anti-CD38 daratumumab for newly-diagnosed and relapsed/refractory patients and the anti-CS1 elotuzumab in the relapse setting. These compounds are under investigation in clinical trials to explore their synergy with other anti-MM regimens, both in the front-line and relapse settings. Other antibodies targeting various antigens are under evaluation. B cell maturation antigens (BCMAs), selectively expressed on plasma cells, emerged as a promising target and several compounds targeting it have been developed. Encouraging results have been reported with antibody drug conjugates (e.g., GSK2857916) and bispecific T cell engagers (BiTEs^®^), including AMG420, which re-directs T cell-mediated cytotoxicity against MM cells. Here, we present an overview on mAbs currently approved for the treatment of MM and promising compounds under investigation.

## 1. Introduction

Multiple myeloma (MM) is a hematologic malignancy characterized by a clonal expansion of aberrant plasma cells in the bone marrow inducing bone lesions, anemia, renal insufficiency and hypercalcemia. In the last two decades, the treatment armamentarium of effective anti-myeloma drugs, used both at diagnosis and at relapse, has been significantly expanded with various compounds of different drug-classes. However, despite the availability of several treatment options, MM still remains an incurable disease whose natural history is characterized by phases of disease remission followed by relapses. The remission duration tends to progressively decrease at every subsequent relapse and MM inevitably becomes refractory to all available agents. Therefore, even if the survival of MM patients, both young and elderly, has steadily increased over time, to date, roughly 50% of patients are alive at 5 years after diagnosis [1,2,3].

With the introduction of effective novel agent combinations, based on immunomodulatory agents (IMiDs) and proteasome inhibitors (PIs), the treatment goal for first-line therapies is now the achievement of minimal residual disease (MRD) negativity [4], which is currently reported in 50–80% of transplant-eligible [5,6,7,8] and in 15–30% of transplant-ineligible patients [9,10,11]. A large meta-analysis demonstrated that reaching MRD negativity (though with some variability in terms of methods and cut-offs adopted) significantly prolonged progression-free survival (PFS) and overall survival (OS) as compared to a MRD-positive status [12]. For this reason, efforts should be made to improve the effectiveness of first-line therapies in inducing deep and durable responses. Regardless of the effectiveness of newer combinations available at diagnosis, the prognosis of high-risk patients (e.g., patients with unfavorable genetics or molecular abnormalities, International Staging System (ISS) stage III, extramedullary disease, or those who experience an early relapse after first-line therapies) is dismal compared to that of standard-risk patients. This evidence prompts the development of different strategies and the adoption of newer drugs in this population, currently representing an unmet medical need.

Furthermore, despite the depth of response obtained with first-line therapies and the duration of the remission, relapse is inevitable in almost all patients with MM, who progressively become refractory to all approved drugs, particularly to IMiDs and PIs. The development of compounds with different mechanisms of action, aiming at synergizing with currently used agents and overcoming drug-induced resistance, is therefore a priority.

Immunotherapy, either passive—with monoclonal antibodies (mAbs) or cellular products directed against neoplastic cells—or active—when the patient’s immune system is stimulated to mount an immune response against tumor cells—represents a pivotal strategy for the treatment of both solid and hematologic malignancies.

MAbs have entered the clinical practice for the treatment of MM [13]. They are selective compounds targeting surface antigens that are highly expressed on aberrant plasma cells and not (or at low density) on normal tissues, thus promoting on-target activity while limiting off-target toxicity. MAbs elicit their therapeutic actions through different mechanisms, including a direct cytotoxicity on the neoplastic cell and immune-mediated mechanisms such as antibody-dependent cell-mediated cytotoxicity (ADCC), antibody-dependent cell-mediated phagocytosis (ADCP) and complement-dependent cytotoxicity (CDC). Monoclonal antibodies can also be exploited to directly target the myeloma cell while conveying a cytotoxic agent, as in the case of antibody-drug conjugates (ADCs), or to engage and activate T cells against the myeloma cell as with bispecific T cell engagers.

Several potential targets have been identified on the myeloma cells and likewise constructs have been designed and tested in MM patients, some of them having already entered the clinical practice.

This review focuses on the strength and controversies of the current treatment strategies exploiting mAbs in MM, as well as on newer experimental immunotherapeutic approaches such as ADCs and bispecific T-cell engagers (BiTEs^®^).

## 2. Monoclonal Antibodies

### 2.1. Anti-CD38 Monoclonal Antibodies

#### 2.1.1. Rationale

CD38 is a transmembrane type II glycoprotein that is highly expressed on normal plasma cells as well as on MM cells [14]. CD38 is also present at lower levels on normal lymphoid and myeloid cells, on red blood cells, as well as on solid tissues such as muscle cells (especially in the airway system), epithelial cells in the prostate and pancreatic beta cells. CD38 acts as a receptor, as an adhesion molecule, and as an ectoenzyme [15,16,17].

**Figure 1:**
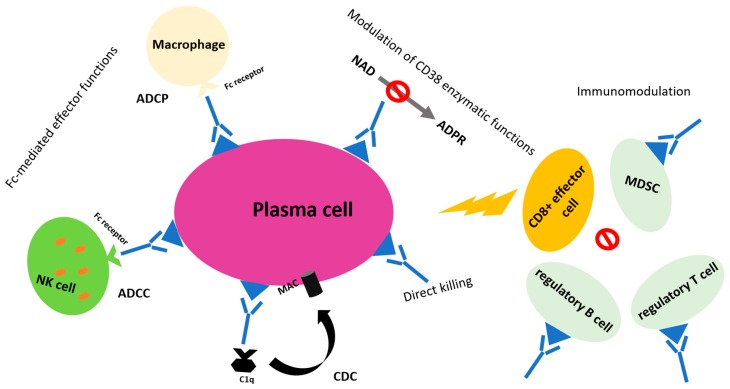
Main mechanisms of action of anti-CD38 monoclonal antibodies. Abbreviations: MDSC, myeloid-derived suppressor cell; ADCC, antibody-dependent cell-mediated cytotoxicity; CDC, complement-dependent cytotoxicity; NAD, nicotinamide adenine dinucleotide; ADPR, adenosine ribose; MAC, membrane attack complex.

Anti-CD38 mAbs elicit their action targeting CD38+ MM cells and inducing effector mechanisms such as ADCC (which relies mainly on natural killer [NK] cells), ADCP, and CDC [18,19,20,21]. An in vitro comparison between the different anti-CD38 molecules showed that ADCC was equally induced by all of them, whereas daratumumab induced the highest CDC at low concentration and ADCP [22]. Alongside immune-mediated cytotoxicity, anti-CD38 mAbs have an immunomodulatory activity that relies on the modulation of immune cells. Myeloid-derived suppressor cells (MDSC), regulatory B cells (Bregs, which promote tumor growth and immune escape), as well as a subset of regulatory T cells (Tregs) express CD38, and their levels are reduced after daratumumab exposure. Conversely, daratumumab results in significant expansion of CD8+ cytotoxic and CD4+ helper T cells, likely following the depletion of regulatory cells. Remarkably, expanded effector T cells also show increased killing capacity due to augmented levels of granzyme B, which activates caspases and triggers cell apoptosis [23,24,25]. In addition, among the activities promoted by CD38, there is a nicotinamide adenine dinucleotide (NAD)-ase activity, which results in reduced levels of NAD+ in T cells, responsible for the loss of their effector functions (exhausted T cells). In murine models, anti-CD38 mAbs administration induced higher levels of NAD+ in T effector cells, thus enhancing their antitumor activity [23]. The main mechanisms of action of anti-CD38 monoclonal antibodies are summarized in Figure 1.

In vitro studies showed a marked synergism between anti-CD38 mAbs (daratumumab, isatuximab and MOR202) and IMiDs (lenalidomide and pomalidomide), mainly owing to an enhanced NK activity elicited by IMiDs that increases both number and activity of NK cells and consequently ADCC, as well as the cytotoxic activity of macrophages, thus stimulating ADCP [26]. This evidence prompted the investigation of the in vivo effect of the addition of anti-CD38 mAbs to IMiD-based combinations. Anti-CD38 mAbs also show an additive effect with PIs [27], although the exact mechanisms are less clear.

#### 2.1.2. Clinical Development

##### Daratumumab

Daratumumab was the first fully human anti-CD38 mAb to be tested in clinical trials. Results of the main clinical trials are summarized in Table 1.

In the phase I GEN501 study, which investigated different doses of daratumumab in relapsed/refractory (RR)MM patients, the greatest activity was reported at the dose of 16 mg/kg, at which 36% of patients achieved a partial response (PR) or better. These results were confirmed by the phase II SIRIUS trial, which reported a 29% overall response rate (ORR) in heavily pretreated patients, resulting into median PFS and OS of 3.7 and 17.5 months, respectively [41]. These results led to the approval, by both the Food and Drug Administration (FDA) and the European Medicines Agency (EMA), of daratumumab as single agent for RRMM patients with 3 prior lines of therapy including a PI and an IMiD.

The synergism showed in vitro by daratumumab and lenalidomide was first translated into a marked in vivo activity of the 3-drug combination daratumumab-lenalidomide-dexamethasone (Dara-Rd) observed in RRMM patients enrolled in the phase II GEN503 study and then confirmed by the phase III POLLUX study. In the POLLUX trial, 569 RRMM patients were randomized to receive standard lenalidomide-dexamethasone (Rd) versus Dara-Rd until disease progression or intolerance [29]. ORR was higher in the triplet arm (93% vs. 76%) as well as the rate of patients achieving minimal residual disease (MRD) negativity (26% vs. 6% of patients, threshold 10^−5^). Median PFS was not reached (NR) versus 17.5 months (hazard ratio [HR] 0.41, *p* < 0.001) in the Dara-Rd versus Rd arms; this benefit was also consistent in patients with high-risk cytogenetics (HR 0.53, *p* = 0.09) [42]. Of notice, the addition of daratumumab to Rd did not significantly increase the rates of grade 3–4 toxicities, with the exception of neutropenia (54% vs. 39%) and infections (28.3% vs. 22.8%). These data supported the approval of Dara-Rd for the treatment of MM patients who had previously received at least 1 line of therapy.

Daratumumab was then evaluated with pomalidomide and dexamethasone (Dara-Pd). In a preliminary phase II trial, this 3-drug regimen showed, in a heavily pretreated population (the median number of prior therapies was 4), ORR (60%) and median PFS (8.8 months) that compared favorably with those of Pd alone (ORR 31%, median PFS 3.8 months) despite the limitations of a cross-trial comparison [32,43]. Following the results of this study, the triplet Dara-Pd received accelerated approval by the FDA for RRMM patients who previously received both an IMiD and a PI. This combination is appealing considering that, in the near future, the majority of newly diagnosed (ND)MM patients will become refractory to continuous lenalidomide after their first line of therapy. Definitive results will come from the phase III trial APOLLO (NCT03180736) comparing Dara-Pd vs. Pd in RRMM patients.

Daratumumab has also been associated with PIs. The phase III CASTOR trial compared bortezomib-dexamethasone (Vd) administered for 8 cycles to daratumumab-Vd (Dara-Vd) for 8 cycles, followed by monthly daratumumab until progression in RRMM patients [30]. The addition of daratumumab resulted in higher ORR (83% vs. 63%) and MRD negativity rate (12% vs. 2%, threshold 10^−5^), and in prolonged PFS (median, 16.7 vs. 7.1 months; HR 0.31; *p* < 0.0001) [31]. Importantly, the MRD negativity rate continued to increase over time for patients receiving Dara-Vd as compared to those receiving Vd, thus highlighting the benefit of continuous treatment with daratumumab. The PFS advantage was also consistent for patients previously exposed to bortezomib (HR 0.35, *p* < 0.001) and for patients with high-risk cytogenetic features detected by fluorescence in situ hybridization (FISH, HR 0.45, *p* = 0.05). The triplet Dara-Vd is currently approved by the FDA and EMA for RRMM patients.

A phase Ib study with carfilzomib-dexamethasone-daratumumab (KdD) induced an objective response in 84% of RRMM patients after both lenalidomide and bortezomib [33]. It was recently announced that the phase III CANDOR study (NCT03158688) comparing Kd to KdD met its primary endpoint, with a 37% reduction in the risk of progression or death (HR 0.63, 95% CI 0.464–0.854, *p* = 0.0014) in patients receiving daratumumab [44].

Because CD38 expression is higher in the early stages of the disease, and mAbs greatly rely on the immune system to exploit their anti-MM activity, it seems reasonable to expect that moving daratumumab to the first-line setting, when the immune-system of a treatment-naïve patient is less compromised, could increase its efficacy. In older patients with newly diagnosed (ND)MM, daratumumab plus bortezomib-melphalan-prednisone (Dara-VMP), followed by daratumumab maintenance, significantly increased the MRD negativity rate as compared to the standard of care VMP (22% vs. 6%, *p* < 0.001, threshold 10^−5^), ultimately prolonging the median PFS (NR after a median follow-up of 17 months vs. 18.1 months, HR 0.50, *p* < 0.001) [11]. Highlighting the role of continuous treatment, a substantial benefit in PFS was detected during the maintenance phase when a lower rate of relapses was observed in patients receiving daratumumab compared with observation (sustained response after 18 months: 77% vs. 60%). This evidence supports the benefit of continuous therapy with daratumumab, which allows better disease control over time compared to fixed duration treatment. A longer follow-up is needed to detect an OS benefit. Dara-VMP has recently been approved by both the FDA and EMA, thus becoming one of the standards of care for transplant-ineligible patients. Impressive results in terms of higher MRD negativity rates (24.2% vs. 7.3%, respectively; *p* < 0.001, threshold 10^−5^) and reduced risk of progression or death (median NR vs. 32 months after a median follow-up of 28 months, HR 0.56, *p* < 0.001) were observed when Dara-Rd was compared to Rd in NDMM patients not suitable for autologous stem-cell transplantation (ASCT; MAIA study [38]). In both Dara-VMP and Dara-Rd regimens, the addition of daratumumab did not negatively affect the safety profiles of VMP and Rd, despite a higher rate of grade 3–4 infections being reported in both studies in patients receiving daratumumab (Dara-VMP 23.1% vs. VMP 14.7%; Dara-Rd 32% vs. Rd 23%); also, the frequency of grade 3–4 neutropenia was higher in patients receiving daratumumab in the MAIA study (50% vs. 35%).

Daratumumab has also been incorporated in the induction, consolidation, and maintenance approach in combination with standard triplets such as bortezomib-thalidomide-dexamethasone (VTd) and bortezomib-lenalidomide-dexamethasone (VRd) as initial treatment for NDMM patients eligible for high-dose melphalan and ASCT.

The phase III CASSIOPEIA trial randomized 1085 transplant-eligible patients to VTd with or without daratumumab as induction and consolidation, followed by daratumumab maintenance or no maintenance. After the consolidation phase, the proportion of MRD-negative patients was higher in the Dara-VTd group than in the VTd group (64% vs. 44%, *p* < 0.001, threshold 10^−5^). This translated into a significantly reduced risk of progression or death in the Dara-VTd arm as compared to the control group (HR for PFS 0.47, *p* < 0.001) [39]. The higher MRD negativity rate reported with daratumumab was also confirmed in ISS-III and high-risk FISH patients (64% vs. 46%, *p* = 0.01; 60% vs. 40%, *p* = 0.06, respectively), with a trend towards PFS improvement with daratumumab (HR 0.66, 95% CI 0.31–1.39; HR 0.67, 95% CI 0.35–1.30, respectively) in these subsets of patients that traditionally represent unmet clinical needs [45]. Mobilization and stem collection after a more intensified induction including daratumumab were adequate. Although patients in the Dara-VTd arm required the use of plerixafor more frequently (22% vs. 8%) and collected less CD34+ cells (median 6.3 × 10^6^/kg vs. 8 × 10^6^/kg), successful ASCT and hematopoietic reconstitution were not affected. Data on maintenance are eagerly awaited. Following the results of the CASSIOPEIA trial, in September 2019, the FDA has approved frontline Dara-VTd as induction for transplant-eligible patients. VRd ± daratumumab as induction and post-ASCT consolidation followed by lenalidomide ± daratumumab maintenance is being compared in the ongoing phase II GRIFFIN trial [40]. The quadruplet significantly improved the MRD negativity rate (threshold 10^−5^) at the end of consolidation, as compared to VRd (47.9% vs. 17.9%, HR 0.23, *p* < 0.001). In both trials, patients treated with daratumumab experienced no significant increase in grade 3–4 non-hematologic adverse events (AEs). Data on maintenance will shed light on the role of daratumumab maintenance, either alone or in combination with lenalidomide.

Other ongoing phase II/III trials evaluating front-line daratumumab in ASCT-eligible patients include the EMN17/PERSEUS trial, which explores the addition of daratumumab to VRd as induction and consolidation and to lenalidomide as maintenance treatment, and the EMN18 study, which compares induction and consolidation with daratumumab-bortezomib-cyclophosphamide-dexamethasone (Dara-VCd) to standard VTd followed by ASCT and maintenance with ixazomib ± daratumumab [46]. The main ongoing trials are summarized in Table 2.

It is currently a matter of debate whether patients with smoldering (S)MM should receive therapy with the aim of preventing the progression to symptomatic MM and the associated morbidity. Two randomized trials demonstrated the benefit of lenalidomide, with or without dexamethasone, in delaying the time to progression to active MM versus observation; importantly, the longer follow-up of the Spanish trial allowed for the detection of an OS advantage for lenalidomide-treated patients [51]. In this setting, a highly targeted therapy with a good safety profile stands out as an ideal option. In the phase II CENTAURUS trial, single-agent daratumumab resulted in an ORR of 56% in high-risk SMM patients, and median PFS was NR after a median follow-up of 26 months [52,53]. The randomized phase III AQUILA study is currently comparing daratumumab administered for 3 years versus standard observation in high-risk SMM (NCT03301220).

One of the limitations to the use of daratumumab is its long infusion time (3.5 h). To deal with this issue, a shorter infusion schedule was tested—daratumumab was administered over a 90 min infusion at the usual dose (16 mg/kg) from the third infusion onward, without increasing the risk for infusion-related reactions (IRRs) or further short-term AEs [54]. A game changer in this setting will be the possibility of delivering daratumumab subcutaneously over a short period of time. The PAVO study explored subcutaneous daratumumab in combination with the recombinant human hyaluronidase PH20 enzyme (rHuPH20), which allowed for the reaching and maintaining of a high-serum concentration of the mAb [55]. At the end of phase Ib of the study, a flat dose of 1800 mg was recommended on the basis of pharmacokinetics, safety (all-grade IRRs 25%), and efficacy data (ORR 42%).

##### Isatuximab

Isatuximab (SAR 650984) is an anti-CD38 immunoglobulin G (IgG)-k chimeric monoclonal antibody that, besides having the same mechanisms of action of daratumumab, holds a unique direct proapoptotic effect independent from the Fc cross-linking [56,57]. Results of the main clinical trials are summarized in Table 1.

Similarly to daratumumab, isatuximab showed a promising activity when administered as a single agent in heavily pre-treated MM patients [58] and has therefore been combined with different anti-MM compounds. A phase Ib trial combined isatuximab at different dose levels with Rd in heavily pretreated MM patients (5 median prior lines of therapy), of whom 68% had already received carfilzomib or pomalidomide and 82% were refractory to lenalidomide. ORR was 51% (and 52% in lenalidomide-refractory patients) and median PFS was 8.5 months. IRRs were the most common AEs related to isatuximab (56% of patients, mainly of grades 1–2 and limited to first infusions) [34]. Another phase Ib trial combined isatuximab with Pd in relapsed patients (3 median prior lines of therapy)—the ORR was 62% and the median PFS was 17.6 months [35]. For both combinations, the selected dose of isatuximab was 10 mg/kg for 4 weekly doses and every 2 weeks thereafter. Of notice, preliminary results of a phase Ib trial in which isatuximab was combined to Kd showed a promising 66% ORR [36].

The ongoing phase III ICARIA trial (NCT02990338) is comparing the triplet isatuximab-pomalidomide-dexamethasone (Isa-Pd) to Pd in 307 RRMM patients who had received at least 2 previous lines of therapy (median lines: 3 in both groups). After a median follow-up of 11.6 months, a consistent benefit in terms of ORR (60% vs. 35%, *p* < 0.001) and PFS (median PFS 11.5 vs. 6.5, HR 0.59, *p* = 0.001) for the triplet arm compared to the control group was shown. Subgroup analysis revealed that PFS benefit was also maintained in high-risk patients (median PFS 7.5 vs. 3.7 months, HR 0.66, 95% CI 0.30–1.28). The median OS was NR in either group, although a trend to improved OS was observed in the triplet arm (HR 0.687, 95% CI 0.461–1.023; *p* = 0.06) [37]. Regarding the safety profile, Isa-Pd induced a slightly higher rate of grade 3–4 infections (42.8% vs. 30.2%) and neutropenia (84.9% vs. 70.1%) [50]. The ongoing phase III IKEMA trial is evaluating the combination of isatuximab with Kd in RRMM patients (NCT03275285).

In transplant-ineligible NDMM patients, isatuximab (10 mg/kg) is being evaluated in a phase Ib trial in combination with VRd as induction (4 cycles) followed by maintenance with Isa-Rd. Preliminary results showed an ORR of 93%, with 38.5% of patients achieving MRD negativity [47]. Another phase Ib trial is evaluating induction with 12 cycles of isatuximab (10/20 mg/kg) plus bortezomib-cyclophosphamide-dexamethasone (VCd), followed by maintenance with single-agent isatuximab in a similar patient population. The ORR was 87%, whereas data on MRD status and PFS are not yet available [48].

In order to improve the poor prognosis of high-risk patients, a phase Ib trial that was specifically designed for high-risk NDMM patients is currently testing a quadruplet regimen combining isatuximab-carfilzomib-lenalidomide-dexamethasone (GMMG-CONCEPT trial [59]).

Finally, the phase III IMROZ study is currently comparing the quadruplet isatuximab-VRd (Isa-VRd) to VRd as upfront treatment for transplant-ineligible patients (NCT03319667). Another ongoing trial is comparing the quadruplet Isa-VRd to isatuximab-VCd (Isa-VCd) in transplant-ineligible patients at diagnosis (NCT02513186).

##### MOR202 and TAK-079

MOR202 and TAK-079 are two anti-CD38 mAbs under development. In preliminary trials, MOR202 proved to be effective in combination with IMiDs; as expected, as this agent does not seem to induce CDC, a low rate of IRRs was observed (10%) [60,61]. In detail, the ORR was 28% in patients receiving MOR202 plus dexamethasone, which increased up to 65% in those receiving MOR202 plus Rd and to 43% in those receiving MOR202 with Pd. However, further development of MOR202 has been discontinued in the United States and Europe. Subcutaneous TAK-079 is currently being tested in preliminary clinical trials on RRMM patients as monotherapy (NCT03439280) and in combination with standard regimens Rd or VRd (NCT03984097). We still need to further define the role of newer anti-CD38 mAbs in the treatment scenario for MM, where daratumumab and isatuximab have proven high efficacy and manageability.

### 2.2. Anti-Signaling Lymphocytic Activation Molecule Family 7 (SLAMF7) Monoclonal Antibodies

#### 2.2.1. Rationale

Signaling lymphocytic activation molecule family 7 (SLAMF7 or CS1) is a cell surface glycoprotein whose expression is essentially restricted to NK cells and both normal and abnormal plasma cells, with 95% of myeloma plasma cells being SLAMF7-positive [62]. In plasma cells and MM cells, the SLAMF7 pathway promotes cell growth and survival, as well as the interaction with the bone marrow micro-environment. Its highly selective expression on plasma cells makes SLAMF7 an optimal target for mAbs.

#### 2.2.2. Clinical Development

Elotuzumab is a humanized IgG-1 monoclonal antibody targeting SLAMF7 that promotes NK-mediated ADCC, directly activates NK cells and interferes with the MM cell adhesion to the bone marrow stromal cells [63,64,65]. Elotuzumab showed no clinically meaningful activity when administered as a single agent—in a phase I dose-escalating study, the best response achieved by RRMM patients treated at different doses of elotuzumab was stable disease (SD, 26%) [66] (Table 3).

Preclinical data showed a synergistic activity of elotuzumab with IMiDs, the latter altering cytokine production and enhancing the activity of NK cells, the main target of elotuzumab immune activity. Promising results in terms of efficacy and tolerability were observed combining elotuzumab with Rd in phase I and II studies, thus providing the rationale for the phase III study ELOQUENT-2, which compared elotuzumab-Rd (Elo-Rd) to Rd in RRMM patients that were not refractory to lenalidomide [67,68,72]. In this study, elotuzumab was administered at the dose of 10 mg/kg and treatment was continued until progression or intolerance [66]. The triplet regimen containing elotuzumab proved to be more effective than Rd in terms of both PFS (19.4 vs. 14.9 months, HR 0.70, *p* < 0.001) and OS (48 vs. 40 months), without adding significant toxicity [73,74]. Patients at first relapse after a remission duration >3.5 years obtained the greater PFS advantage with Elo-Rd [75], showing that the greatest benefit with Elo-Rd could be obtained in patients with a slow and indolent progression. Elo-Rd is currently approved by both the FDA and EMA for the treatment of RRMM patients after 1 line of therapy.

The synergistic activity between elotuzumab and IMiDs prompted the investigators to test elotuzumab both in the upfront setting in combination with lenalidomide and at relapse with the third-generation IMiD pomalidomide [76]. The ongoing phase III study ELOQUENT-1, whose results are not yet available, enrolled NDMM patients ineligible for high-dose melphalan and ASCT in order to investigate the benefit of the addition of elotuzumab to the standard doublet Rd, possibly establishing a new standard of care in this setting.

In the randomized phase II ELOQUENT-3 trial, the addition of elotuzumab to Pd in RRMM patients significantly increased the ORR (53% vs. 26%) and prolonged median PFS (10.3 vs. 4.7 months, HR 0.54, *p* = 0.008), as compared to Pd alone. Again, the safety profiles of the two arms of the study were overlapping, meaning that elotuzumab did not add significant toxicity to Pd [69]. On this basis, in 2018 the FDA approved the triplet elotuzumab-Pd for the treatment of RRMM patients who had received at least 2 prior regimens including lenalidomide and a PI.

In preclinical models, elotuzumab activity was potentiated by bortezomib, which makes myeloma cells more vulnerable to NK-mediated lysis [77]. This combination was subsequently tested in clinical trials. In a phase I study on RRMM patients, elotuzumab was combined with bortezomib, showing an ORR of 48% and a median time to progression of 9.5 months [70]. The triplet elotuzumab-Vd (Elo-Vd, with elotuzumab administered at 10 mg/kg) was subsequently compared to Vd in a phase II trial on 152 RRMM patients, half of which had already received bortezomib in previous lines of therapy. ORR was similar between the two groups (66% vs. 63%), and a slight PFS advantage was observed in the triplet arm that nonetheless did not reach statistical significance (median, 9.7 vs. 6.9 months, HR 0.72, *p* = 0.09) [71]. The most common grade ≥3 AEs were infections (Elo-Vd 21% vs. Vd 13%) and thrombocytopenia (Elo-Vd 9% vs. Vd 17%). Because elotuzumab elicits its action by binding its Fc portion to the Fc gamma receptor III on NK cells, different allelic variants of the receptor were analyzed to evaluate possible predictors of elotuzumab efficacy. In this study, patients homozygous for the high-affinity Fc gamma receptor IIIa (FcγRIIIa) V allele showed longer PFS as compared to patients homozygous for the low-affinity allele. Considering the number of treatment options currently approved, the availability of a predictor of response could help clinicians in the choice of the most appropriate treatment.

Elotuzumab-Rd has also been investigated as a prevention strategy in high-risk SMM. In a phase II study (NCT02279394), patients received 8 cycles of elotuzumab-Rd and were subsequently allowed to continue with elotuzumab and lenalidomide maintenance until progression to symptomatic MM [78]. Preliminary data showed an ORR of 84% with no patients progressing at MM at the present follow-up of 29 months. Grade 3–4 toxicities included neutropenia (16%) and infections (12%), mainly related to lenalidomide. Again, single-agent elotuzumab did not show any clinical activity when in the setting of SMM [79].

Of interest, the rate of IRRs observed with elotuzumab—which were mostly mild in nature (grades 1–2) and rarely leading to treatment discontinuation—was definitely lower (10%) than that observed with other mAbs, making elotuzumab-based combinations appealing options for the treatment of frail patients [80].

Numerous studies are currently ongoing with elotuzumab-based combinations, such as elotuzumab-VRd (Elo-VRd, NCT02375555), elotuzumab-KRd (Elo-KRd, NCT02969837) and elotuzumab plus pomalidomide-bortezomib-dexamethasone (NCT02718833).

### 2.3. Anti-Programmed Death 1 (PD-1) Monoclonal Antibodies

#### 2.3.1. Rationale

The programmed death 1 (PD-1) receptor is a transmembrane glycoprotein expressed on antigen-activated T cells and B cells. The binding of PD-1 ligands (PD-1-L1 and PD-1-L2) on PD-1 receptor results in the downregulation of immune T cell functions [81]. Preclinical data showed that PD-1/L1 is highly expressed on myeloma cells and, at variable levels, on normal plasma cells. It is also expressed at high levels on dendritic cells in the myeloma microenvironment [82,83]. Moreover, T cells derived from myeloma patients showed higher rates of PD-1 expression as compared to T cells from healthy donors, suggesting that the PD-1/PD-L1 pathway plays an important role in the immune escape of myeloma cells. Given these premises, targeting PD-1 and PD-L1 with monoclonal antibodies seems to be a promising strategy for the treatment of MM.

#### 2.3.2. Clinical Development

Monoclonal antibodies directed against the PD-1/PD-L1 pathway can be divided into molecules targeting PD-1 (e.g., pembrolizumab and cemiplimab) and molecules targeting PD-L1 (e.g., durvalumab). Pembrolizumab monotherapy did not show efficacy as a single agent in 30 heavily pretreated myeloma patients (4 median prior lines of therapy) [84]. Pembrolizumab was subsequently combined with immunomodulatory agents, as preclinical data suggested that IMiDs could contribute to the downregulation of the PD-1/PD-L1 pathway [85]. In phase II trials, ORR was 50% in RRMM patients receiving pembrolizumab plus Rd and 60% in patients receiving pembrolizumab plus Pd [86]. However, in 2017, following the preliminary results of the two randomized phase III trials KEYNOTE 185 (pembrolizumab-Rd vs. Rd) and KEYNOTE 183 (pembrolizumab-Pd vs. Pd), the FDA prompted the discontinuation of any further investigations of these combinations, in light of the increased risk of death for patients in the pembrolizumab group versus the control group (HR for OS in pembrolizumab-Pd vs. Pd 1.61; HR for OS in pembrolizumab-Rd vs. Rd 2.06) [87,88]. The main concern with this combination is indeed the increased risk of enhancing immune-mediated toxicity, resulting in various AEs, such as dermatologic, pulmonary, cardiac, gastrointestinal and hepatic toxicities. These results questioned the utility of anti-PD-1 mAbs in MM, at least in combination with IMiDs. Different molecules are currently under evaluation in combination with other agents. The anti-PD-1 cemiplimab is being evaluated in a phase I/II trial in combination with isatuximab (NCT03194867), whereas durvalumab is being tested in combination with daratumumab (NCT03000452). However, the future role of this class of molecules in the treatment of MM remains debated.

## 3. Antibody Drug Conjugates

### 3.1. Rationale

Antibody drug conjugates (ADCs) are monoclonal antibodies bound by a chemical linker to a cytotoxic compound directed against surface antigens of the targeted cells. ADCs selectively target cells expressing their target antigen and are then internalized releasing the cytotoxic component through lysosome degradation, causing cell death. This targeted delivery limits the systemic exposure to the cytotoxic compound, sparing the non-malignant cells and tissues that do not express the target antigen, consequently limiting its off-target toxic effects [89,90]. In the past few years, interest has been raised around ADCs for the treatment of lymphoid malignancies, with brentuximab vedotin being the first agent of this class to receive FDA and EMA approval for the treatment of relapsed/refractory Hodgkin lymphoma and anaplastic large cell lymphoma in 2011–2012 [91,92]. In MM, ADCs showed preclinical activity in in vitro and in xenograft models and are currently under evaluation in clinical trials for relapsed MM patients [93,94,95]. One of the main challenges with ADCs is the choice of the most appropriate surface antigens to be targeted, which should be highly expressed only on malignant cells and not on normal tissues. Several target antigens have been identified on plasma cells: CD56, CD138, CD74, Fc receptor-like 5 and B cell maturation antigen (BCMA) [96]; of these, CD56 is expressed only on MM cells, with no expression on normal plasma cells, whereas other antigens are expressed on both malignant and non-malignant plasma cells, although at different levels [97]. The cytotoxic compound is typically a small molecular weight toxin with potent activity at low concentrations. Such molecules, usually not employed for systemic chemotherapy due to their excessive toxicity, can cause cell death due to two different mechanisms: cell cycle interference through microtubules inhibitions and DNA damage. Maytansinoid derivatives are microtubule inhibitors, including DM1 (emtansine and mertansine), DM4 (soravtansine and ravtansine) and auristatin derivatives (including monomethyl-auristatin E (MMAE, vedotin) and monomethyl auristatin F (MMAF, mafodotin)) [98,99,100,101]. Calicheamicins, duocarymycins and pyrrolobenzodiazepine dimmers are DNA-damaging agents [102,103].

### 3.2. Clinical Development

Table 4 summarizes the results of the main studies with ADCs in MM.

In 2018, the results of a first in-human phase I study investigating GSK2857916, a BCMA-targeting mAb conjugated to the antimitotic agent monomethyl auristatin F (MMAF), in 73 RRMM patients were published. BCMA, a transmembrane receptor required for B cell maturation, was chosen as an optimal target, as it is expressed almost exclusively on MM cells and plasma cells [104,105,106]. In the dose-escalation phase of the study, 38 patients received escalating doses of IV GSK2857916 (0.03–4.6 mg/kg) every 3 weeks. In the dose-expansion phase of the study, 35 patients received the recommended phase II dose of GSK2857916 (3.4 mg/kg) every 3 weeks until progression. Among heavily pre-treated patients, GSK2857916 induced an objective response in 60% of them, with 15% of patients achieving a CR or a stringent CR (sCR). Remarkably, the ORR in patients previously treated with anti-CD38 mAbs and refractory to both IMiDs and PIs was 38%. Responses were rapid (median time to response 1.2 months) and durable (median duration of response 14.3 months). Overall, median PFS was 12 months; median PFS was 7.9 months in double-refractory patients (to IMiDs and PIs) and 6.2 months in double-refractory patients with prior daratumumab. The most common treatment-related toxicities were thrombocytopenia (63%; grades 3–4: 26%) and corneal events in terms of blurred vision and photophobia (51%; grades 3–4: 3%). Ocular toxicity was mainly limited to grades 1–2 and was reversible and easily manageable with dose reductions (51% of patients) [104,106]. Because GSK285791 showed high ORR in patients previously treated with anti-CD38 mAbs, a phase I/II clinical trial exploring its efficacy as monotherapy in patients with previous exposure to daratumumab/isatuximab has recently completed enrollment and results will soon be available (NCT03525678). Ongoing trials are evaluating its safety and efficacy in combination with pembrolizumab (NCT03848845), pomalidomide (NCT03715478), and lenalidomide versus bortezomib (NCT03544281).

Indatuximab-ravtansine (BT062) is an anti-CD138 IgG4 monoclonal antibody that delivers the microtubule inhibitor maytansinoid ravtansine to CD138-positive cells. CD138 is a transmembrane protein receptor upregulated by myeloma cells. BT062 monotherapy was evaluated in 67 heavily pretreated RRMM patients (median previous therapies 7, range 1–15). The most common grade 3–4 toxicities were fatigue (7%), anemia (7%), and diarrhea (4%). At the maximum tolerated dose (MTD) of BT062 (140 mg/m^2^), 62% of patients achieved SD, whereas an objective response was observed in 5% of patients only. Median PFS and OS were 3 and 26 months, respectively [107]. BT062 is currently under evaluation in combination with lenalidomide or pomalidomide plus dexamethasone in RRMM patients. In patients receiving BT062 + lenalidomide (*n* = 47), ORR was 77% and median PFS was 16.4 months, whereas in those receiving the ADC in combination with pomalidomide (*n* = 17) ORR was 79% and median PFS was NR after 7 months of follow-up. These triplets were well tolerated, with main AEs being fatigue and diarrhea [108].

Lorvotuzumab-mertansine (IMGN901) is an anti-CD56 mAb linked to the maytansinoid mertansine, which inhibits microtubules assembly interfering with cell cycle and therefore causing cell death. A phase I trial enrolling 37 heavily pre-treated patients (78% had ≥3 lines of therapy) with CD56+ RRMM explored the safety and efficacy of single-agent IMGN901. The MTD was established at 112 mg/m^2^. Forty-three percent of patients experienced SD, 6% PR, and no patient reached a very good (VG)PR or better, with a median PFS of 6.5 months. The toxicity profile was manageable and drug discontinuation due to AEs was observed in 24% of patients, with peripheral neuropathy (grades 3–4: 5.3%) being the most common toxicity leading to discontinuation [109]. IMGN901 is also being evaluated in combination with Rd. Preliminary reports showed an ORR of 56%, including 2 CRs and 8 VGPRs. The most common toxicity was peripheral neuropathy, although no grade 3–4 events occurred at the MTD of 75 mg/m^2^ [111].

ADCs, particularly GSK285791, displayed a promising efficacy among heavily pre-treated patients. Their unique mechanism of action and preliminary efficacy data make these drugs an appealing treatment option in patients who have become refractory to IMiDs, PIs, and anti-CD38. Furthermore, the lack of cross-resistance with currently approved agents also prompts their investigation in the earlier phase of the disease, such as in the context of a consolidation strategy in high-risk patients or those MRD-positive after the induction/transplant phases.

Other compounds are under preliminary evaluation in MM. CD74, a transmembrane glycoprotein expressed in more than 90% of B cell malignancies, is the target of the ADC milatuzumab-doxorubicin (of hLL1-DOX) [110]. In a preliminary study, the ADC proved to be well tolerated, with SD being the best response achieved (26% patients) with this agent used as monotherapy for RRMM patients [112]. Preclinical results showing synergistic activity of hLL1-DOX with PIs and IMiDs provide the biological rationale for the evaluation of this ADC in combination with other agents.

## 4. Bispecific T Cell Engagers

Bispecific monoclonal antibodies are engineered molecules meant to redirect immune effector cells, mainly T and NK cells, to tumor cells, thus restoring the immune suppressor activity of the immune system against neoplastic cells. Bispecific T cell engager molecules are a class of bispecific antibodies combining the minimal binding domains (variable fragments (Fv), single chains) of two different monoclonal antibodies on one polypeptide chain [113]. They are characterized by a small size, allowing optimal proximity between the engaged T cell and the target tumor cell; for this very reason, they are active at low concentrations, as compared to bispecific antibodies. Bispecific antibodies usually link the invariant part of CD3 of the T cell receptor (TCR) on T cells and a tumor-specific antigen, thus leading to T cell activation and proliferation and tumor cell apoptosis [114]. The first approved bispecific T cell engager was the anti-CD19 blinatumomab for the treatment of RR B cell acute lymphoblastic leukemia [115].

Among potential targets on plasma cells, BCMA, CD38 and SLAMF7 have been chosen to design anti-MM bispecific antibodies [23,116], with BCMA representing the most promising target. Another potential target due to its high expression on PC is G-protein coupled receptor C family 5D (GPRC5D), whose function is still unclear [117,118].

### Clinical Development

AMG420 is an anti-BCMA bispecific T cell engager that is currently being evaluated in the first in-human dose escalation trial enrolling RRMM patients (4 median prior lines of therapy). AMG420 was administered as a continuous intravenous infusion due to its short half-life at doses ranging from 0.2 to 800 mcg/die. At the MTD of 400 mcg/day, the ORR was 70%, with 5/10 patients obtaining MRD-negative sCRs (10^−4^) [119]. Dose-limiting toxicities were cytokine release syndrome (CRS, 1 patient) and peripheral neuropathy (2 patients). Only 1 grade 3 CRS was observed, and no grade 3–4 AEs related to the central nervous system were registered at the MTD. Another anti-BCMA BiTE^®^, AMG 701, has a longer half-life (112 h), thus allowing weekly short-term infusion. AMG 701 is currently being investigated in the first phase I trial [120].

BiTEs^®^ currently under investigation are listed in Table 5.

## 5. Conclusions and Future Directions

Immunotherapy, and in particular mAbs, is no longer an appealing future perspective, but rather a valuable present therapeutic option for MM patients—having demonstrated to induce a response where conventional agents had failed—to increase the depth of response obtained with standard regimens acting in synergy with them and, ultimately, to prolong both PFS and OS. The ‘guiding star’ in this treatment landscape is definitely the anti-CD38 mAb, which rapidly turned from being a valid alternative for RRMM patients without further viable therapeutic options to being the backbone of virtually all present and future combinations adopted as frontline therapies. However, given the different combinations of both daratumumab and isatuximab with backbone therapies (available or under evaluation), we still need to define which anti-CD38 mAb should be used considering the unavailability of data on the superiority of one over the other. Another open issue is as to what could be the effectiveness of re-treatment with the same, or a different, CD38 mAb. Arguably, this last question will be answered in the near future, thanks to the increasing use of anti-CD38 mAb combinations in early lines. These issues are particularly challenging considering the wide heterogeneity of myeloma cell populations [121]. Immunotherapy seems to be a potential strategy for targeting virtually all tumor subclones, as effector mechanisms rely on the patient immune system. Ongoing studies are exploring the different potential mechanisms of resistance to anti-CD38 mAbs, as well as how to overcome them. Lower basal levels of the target antigen have been proposed as a possible mechanism of intrinsic resistance to mAbs [122,123]. Regarding daratumumab, the downregulation of CD38 on cell surfaces could partially explain the loss of response to mAb therapy [124]. Interestingly, myeloma cells exposed to isatuximab and MOR202 did not show such a downregulation [125,126]. An intriguing way to overcome the acquired resistance derived from antigen downregulation could be the addition of molecules able to re-induce CD38 expression on cell surface, such as all-trans retinoic acid (ATRA) or panobinostat [127,128]. Finally, other proposed mechanisms of resistance under evaluation include the modification of the expression of adhesion molecules and the overexpression of complement inhibitors. In the context of the currently available anti-CD38 combinations, the role of anti-SLAMF7 mAb-based combinations is unclear—both anti-CD38 and anti-SLAMF7 antibodies have been combined with the same backbones (Rd, Pd, Vd); both anti-CD38 and anti-SLAMF7 mAbs showed encouraging efficacy data even in high-risk patients, but not substantial enough to suggest an ability to completely overcome their adverse prognoses; and, finally, both mAbs have a very good safety profile. Studies showing the better efficacy of one mAb combination over the other are currently lacking. The role of both mAbs in the treatment of SMM also needs to be defined—their good safety profiles make them good candidates for the treatment of a still asymptomatic disease, but their efficacy and the possibility to improve OS still need to be shown. ADCs and BiTEs^®^ are fascinating constructs potentially able to either carry toxic compounds or redirect T cells against MM cells in a very specific way, thus limiting off-target toxicities. The preliminary results obtained with single-agent ADCs or BiTEs^®^ in heavily pre-treated patients are by far exceeding expectations, especially if compared to the results obtained with the currently available single-agent drugs. Future studies will shed light on their role in the treatment of MM patients and on their efficacy when used earlier in the course of the disease; they will also explore how to improve their feasibility and treatment compliance, especially in relation to the continuous intravenous infusion characteristic of the BiTEs^®^ evaluated in MM thus far. In this field, the compounds showing the most encouraging preclinical results are bispecific antibodies with extended half-life such as the anti-BCMAs AMG701 and PF3135, which would allow a weekly administration [129,130]. Moreover, we still need to decipher the exact mechanisms of resistance and how to revert them, as well as the best drug-partners to enhance their efficacy in different settings. We have to devise the proper antigen selection and payload choice that will be critical for their success in the treatment of MM. MAbs can also be conjugated with radioisotopes in order to increase the antitumor effect of the molecules. Daratumumab has been combined with different radionuclides (e.g., actinium-225), resulting in an increased tumoricidal effect besides its Fc-effector functions in preclinical models [131]. Bispecific pretargeted radiolabeled antibodies showed an even greater biodistribution to tumor cells and, in future, can represent an appealing approach for the treatment of MM, especially for heavily pretreated patients who usually remain sensitive to radiation [132]. Regarding the use of mAbs, another field of interest is the use of radiolabeled antibodies for imaging assessment with immuno-positron emission tomography (immuno-PET) [133]. Indeed, surface antigens expressed on myeloma cells could be a target for radiolabeled mAbs, which would allow highly specific tumor detection and precise response assessment. Daratumumab has already been labeled to different positron emitters showing excellent targeting in preclinical models [134,135,136]. With these premises, immuno-PET could represent a useful tool for imaging assessment and also for guiding treatment strategies, as this technique could potentially be used to predict the effectiveness of mAb therapy.

Another issue is timing, that is to say, the most appropriate phase of treatment or disease in which these different classes of drugs should be used—if at diagnosis, at the evidence of MRD persistence in an effort to eradicate a resistant clone, or at relapse once conventional treatments have failed. In a highly competitive setting, with few validated targets (CS1, CD38, BCMA) and many different technologies (ADC, BiTEs^®^, chimeric antigen receptor [CAR] T cells), both preclinical and clinical studies are critical to identify the most promising compounds. Along with the refinement of the existing drug regimens and treatment strategies and the development of new ones, a better understanding of the role of the immune system in the pathogenesis of MM will certainly be necessary.

## Figures and Tables

**Table 1:** Results of the main clinical trials with anti-CD38 monoclonal antibodies daratumumab and isatuximab.

Study	Phase	Number of Patients	Median Previous Line	Regimen	ORR	Median PFS (Months)	Median OS (Months)
**RELAPSED PATIENTS**							
GEN501 + SIRIUS POOLED [28]	II	148	5	Daratumumab single agent	31.1%	4	20.1
POLLUX [29]	III	569	1	Dara-Rd vs. Rd	92.9% vs. 76.4%	NR vs. 17.5	1-year OS 92.1% vs. 86.8%
CASTOR [30,31]	III	498	2	Dara-Vd vs. Vd	83.8% vs. 63.2%	16.7 vs. 7.1	NA
NCT01998971 [32]	II	103	4	Dara-Poma-dex	60%	8.8	17.5
NCT01998971 [33]	Ib	85	2	Dara-Kd	84%	1-year PFS 74%	1-year OS 82%
NCT01749969 [34]	Ib	57	5	Isa-Rd	56%	8.5	NR
NCT02283775 [35]	Ib	45	3	Isa-Pd	62%	17.6	NR
NCT02332850 [36]	Ib	33	3	Isa-Kd	66%	NR	NR
ICARIA [37]	III	307	3	Isa-Pd vs. Pd	60% vs. 35%	11.5 vs. 6.5	NA
**NEWLY DIAGNOSED PATIENTS**							
ALCYONE [11]	III	706 TNE	−	Dara-VMP vs. VMP	90.9% vs. 73.9%	NR vs. 18.1	NA
MAIA [38]	III	737 TNE	−	Dara-Rd vs. Rd	92.9% vs. 81.3%	NR vs. 31.9	NA
CASSIOPEIA [39]	III	1085 TE	−	Dara-VTd vs. VTd	≥CR 39% vs. 26%	NA	NA
GRIFFIN [40]	II	207 TE	−	Dara-VRd vs VRd	51.5% vs. 42.3%	NA	NA

Abbreviations: ORR, overall response rate; PFS, progression-free survival; OS, overall survival; Dara, daratumumab; Isa, isatuximab; V, bortezomib; C, cyclophosphamide; d, dex, dexamethasone; T, thalidomide; R, lenalidomide; K, carfilzomib; Poma, pomalidomide; M, melphalan; P, prednisone; NR, not reached; NA, not yet available; TNE, transplant ineligible; TE, transplant eligible; CR, complete response.

**Table 2:** Main ongoing trials involving daratumumab and isatuximab in multiple myeloma patients.

Study	Setting	Phase	Study Design
**DARATUMUMAB**			
NCT03710603 [46]	NDMM TE (690 pts)	III	Dara-VRd + ASCT + Dara-VRd consolidation + Dara-R maintenance vs. VRd + ASCT + VRd consolidation + R maintenance
NCT03896737	NDMM TE (≈400 pts)	II	Dara-VCd + double ASCT + Dara-VCd consolidation vs. VTd + double ASCT + VTd consolidation Second randomization: Ixa maintenance vs. Ixa-Dara
NCT03180736	RRMM (302 pts)	III	Dara-Poma-dex vs. Poma-dex
NCT03158688	RRMM (466 pts)	III	Dara-Kd vs. Kd
**ISATUXIMAB**			
NCT02513186 [47,48]	NDMM NTE (88 pts)	I/II	Isa-VCd vs. Isa-VRd
NCT03319667 [49]	NDMM, NTE (475 pts)	III	Isa-VRd vs. VRd
NCT03275285	RRMM (302 pts)	III	Isa-Kd vs. Kd
NCT02990338 [50]	RRMM (300 pts)	III	Isa-Poma-dex vs. Poma-dex

Abbreviations: pts, patients; NDMM, newly diagnosed multiple myeloma; RRMM, relapsed/refractory MM; Dara, daratumumab; Isa, isatuximab; ASCT, autologous stem-cell transplantation; TE, transplant eligible; NTE, transplant ineligible; Ixa, ixazomib; V, bortezomib; C, cyclophosphamide; d, dex, dexamethasone; T, thalidomide; R, lenalidomide; K, carfilzomib; Poma, pomalidomide.

**Table 3:** Results of the main clinical trials with anti-signaling lymphocytic activation molecule family 7 (SLAMF7) monoclonal antibody elotuzumab.

Study	Phase	Number of Patients	Median Previous Line	Regimen	ORR	Median PFS (Months)	Median OS (Months)
NCT00425347 [66]	I	35	5	Elo (0.5–20 mg/kg)	0	NA	NA
ELOQUENT-2 [67,68]	III	321	2	Elo-Rd vs. Rd	79% vs. 66%	19.4 vs.14.9	48 vs. 40
ELOQUENT-3 [69]	II	117	3	Elo-Poma-dex vs. Pd	53%vs.26%	10.3 vs. 4.7	NA
NCT00726869 [70]	I	28	2	Elo-V	48%	9.5	NA
NCT01478048 [71]	II	152	NA	Elo-Vd vs. Vd	66% vs. 63%	9.7 vs. 6.9	2-year OS 73% vs. 66%

Abbreviations: Elo, elotuzumab; ORR, overall response rate; PFS, progression-free survival; OS, overall survival; d, dex, dexamethasone; R, lenalidomide; Poma, pomalidomide; V, bortezomib; NR, not reached; NA, not yet available.

**Table 4:** Results of preliminary clinical trials with antibody-drug conjugates (ADCs).

Study	Phase	ADC	Target	Cytotoxic Agent	Respinse	Key Toxicities (G3–4)
NCT02064387 [104,105,106]	I	GSK2857916	BCMA	MMAF	ORR 60% PFS 12 m	Thrombocyotpenia 35% Corneal events 14%
NCT01001442 [107]	I	Indatuximab-ravtansine	CD138	DM4	ORR 6% PFS 3 m OS 26 m	Fatigue (7%) Anemia (7%) Diarrhea (4%)
NCT01638936 [108]		Indatuximab-ravtansine + Rd or + Poma-dex	CD138	DM4	ORR 77% PFS 16.4 m ORR 79% PFS NR	Diarrhea Fatigue Nausea
NCT00991562 [109]	I	Lorvotuzumab-mertansine	CD56	DM1	ORR 6% PFS 6.5 m	Peripheral neuropathy (5.3%)
NCT01101594 [110]	I	Milatuzumab-doxorubicin	CD74	Doxorubicin	ORR 0%	Anemia (4%) Back pain (4%) CRS (4%)

Abbreviations: R, lenalidomide; d, dex, dexamethasone; ORR, overall response rate; PFS, progression-free survival; OS, overall survival; NR, not reached; CRS, cytokine release syndrome; G, grade; MMAF, monomethyl auristatin F; BCMA, B cell maturation antigen.

**Table 5:** Bispecific T cell-engaging agents (BiTEs^®^) for the treatment of multiple myeloma.

ClinicalTrials.Gov ID	Agent	Target
NCT02514239	AMG 420	BCMA
NCT03287908	AMG 701	BCMA
NCT03486067	CC-93269	BCMA
NCT03145181	JNJ-64007957	BCMA
NCT03269136	PF-06863135	BCMA
NCT03761108	REGN5458	BCMA
NCT03399799	JNJ-64407564	GPRC5D
NCT03309111	GBR 1342	CD38

Abbreviations: BCMA, B cell maturation antigen; CPRC5D, G-protein coupled receptor C family 5D.
